# Tremor in cervical dystonia

**DOI:** 10.3389/dyst.2024.11309

**Published:** 2024-03-21

**Authors:** Sinem Balta Beylergil, Krishna Nikhil Mukunda, Mohamed Elkasaby, Joel S. Perlmutter, Stewart Factor, Tobias Bäumer, Jeanne Feurestein, Erika Shelton, Steven Bellows, Joseph Jankovic, Abhimanyu Mahajan, Tila Wamer-Rosen, Stephen G. Reich, Aparna Wagle Shukla, Irene Malaty, Alberto Espay, Kevin Duque, Mark S. LeDoux, Rachel Saunders-Pullman, Katherine Leaver, Samuel Frank, Alexander Pantelyat, Victor Fung, Sarah Pirio Richardson, Brian Berman, Natividad Stover, Andres Deik, William Ondo, Christopher Groth, Hyder A. Jinnah, Aasef G. Shaikh

**Affiliations:** 1 Department of Neurology, Case Western Reserve University, Cleveland, OH, United States; 2 Department of Neurology and Neurologic Surgery, Washington University School of Medicine, St. Louis, MO, United States; 3 Department of Neurology, Emory University School of Medicine, Atlanta, GA, United States; 4 Institute of Systems Motor Science, University of Lübeck, Lübeck, Germany; 5 Department of Neurology, University of Colorado School of Medicine, Aurora, CO, United States; 6 Department of Neurology, Baylor College of Medicine, Houston, TX, United States; 7 Department of Neurological Sciences, Rush University Medical Center, Chicago, IL, United States; 8 Department of Neurology, University of Maryland School of Medicine, Baltimore, MD, Untied States; 9 Department of Neurology, University of Florida, Gainesville, FL, United States; 10 Deparment of Neurology, University of Cincinnati, Cincinnati, OH, United States; 11 Department of Neurology, University of Tennessee Health Science Center, Memphis, TN, United States; 12 Department of Neurology, Mount Sinai Beth Israel, New York, NY, United States; 13 Department of Neurology, Harvard Medical School, Boston, MA, United States; 14 Department of Neurology, Johns Hopkins University School of Medicine, Baltimore, MD, United States; 15 Sydney Medical School, University of Sydney, Sydney, NSW, Australia; 16 Department of Neurology, University of New Mexico, Albuquerque, NM, United States; 17 Department of Neurology, Virginia Commonwealth University, Richmond, VA, United States; 18 Department of Neurology, The University of Alabama, Tuscaloosa, AL, United States; 19 Department of Neurology, University of Pennsylvania, Philadelphia, PA, United States; 20 Houston Methodist Neurological Institute, Weill Cornell Medical School, Houston, TX, United States; 21 Department of Neurology, University of Iowa, Iowa City, IA, United States

**Keywords:** dystonia, tremor, cervical dystonia, reularity, jerkiness

## Abstract

**Background:**

Cervical dystonia (CD) is the most common form of focal dystonia encountered in the clinic. Approximately one-third of CD patients have co-existing tremor in the head and hands. Assessment of tremor as regular or irregular in context of its oscillation trajectory, frequency, and amplitude is a major clinical challenge and can confound the diagnosis of CD. The misdiagnosis may lead to therapeutic failures, poor quality of life, and poor utilization of medical and financial resources.

**Methods:**

We analyzed the largest cohort of CD patients (*n* = 3117) available to date, collected from 37 movement disorder centers in North America, Europe, and Asia. We used machine learning to determine what clinical features from clinician reports predicted the presence of tremor as well as its regular or irregular appearance.

**Results:**

Out of 3,117 CD patients, 1,367 had neck tremor. The neck tremor was interpreted as irregular in 1,022, regular in 345, and mixed (both irregular and regular) in 442. A feature importance analysis determined that greater severity of CD, longer disease duration, and older age, in descending order, predicted the presence of neck tremor. The probability of neck tremor was reduced if the dystonia affected other body parts in addition to the neck. We also found a significantly heightened risk for developing neck tremor in women. An additional feature importance analysis indicated that increased severity of dystonia affecting other body parts, severity of CD, and prolonged disease duration was associated with a lower likelihood of regular neck tremor while increased age predicted a higher likelihood.

**Conclusion:**

Machine learning recognized the most relevant clinical features that can predict concurrent neck tremor and its irregularity in a large multi-center dystonia cohort. These results may facilitate a more accurate description of neck tremor and improved care path in CD.

## Introduction

**FIGURE 1:**
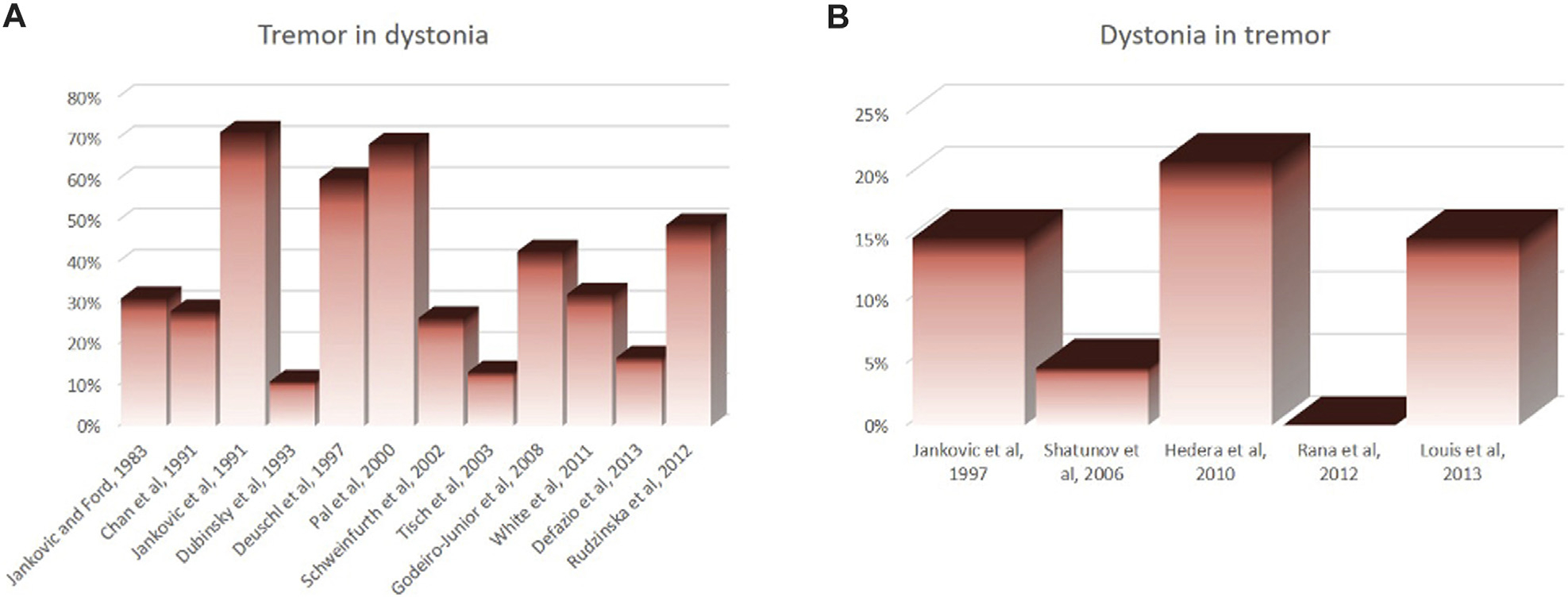
Disparity in reported prevalence rate of tremor in patients who have dystonia **(A)** and prevalence rate of dystonia in those who have tremor **(B)**.

Dystonia and tremor are two distinct neurological signs, which are often present in the same individual and are closely related. Despite this close relationship between the two conditions, previous studies showed highly variable prevalence of tremor in dystonia, ranging from 10% to 70% (Figure 1A). Such disparity in prevalence is also seen for dystonia in those who have tremor, ranging from 0% to 21% (Figure 1B). There have been several attempts to define “tremor-like” dystonic movements. Fahn (1984) called “dystonic tremor” based on its irregularity, jerky appearance of the waveform, dependence on the region of the body affected, and the presence of null point [1, 2]. However, “irregularity” is often viewed as variability in tremor frequency and amplitude, not just the “jerky” shape. On the contrary, the 1998 Movement Disorders Society (MDS) consensus statement on tremor classification [3], tremor is classified as dystonic tremor when it affects a body part that is also affected by dystonia. The 1998 MDS consensus added the definition “tremor associated with dystonia (TAWD)” to this statement to accommodate the cases where tremor occurs in body regions without overt dystonia.

There were a few caveats with the 1998 MDS committee’s definition for dystonic tremor: the requirement of co-existing twisting movements, which can be subjective. For example, while slight tilting of the neck or minor spooning of the fingers are viewed as dystonia by some investigators, these are potentially normal variants of motor behavior according to others [4–12]. The other limitation of the 1998 MDS committee’s definition was that its mutually exclusive diagnostic criteria inherently precluded the possibility that tremor and dystonia may be two distinct disorders that co-occur. The fundamental disagreement on the definition of dystonic tremor called for more general re-evaluation of the operational definitions of how tremor relates to dystonia [4, 13–19]. The 2018 MDS Task Force on Tremor recently retained the definitions of dystonic tremor TAWD [20]. The 2018 taskforce divided essential tremor into essential tremor (i.e., pure tremor) and “essential tremor plus” (i.e., tremor that may be combined with questionable dystonic features) [20, 21].

It is particularly important to understand the relationship of neck tremor and CD because they are most common of all other types of tremor dystonia combinations. CD and jerky repetitive neck movements have different pathophysiological correlates compared to more sinusoidal neck oscillations that appear like tremor seen with essential tremor [22, 23]. To understand the relationships between neck tremor and CD, it is necessary to support the expert consensus-based opinions with empiric evidence. The need is critical from both clinical and research standpoints. A recent study examining a large number of CD cases from multiple centers provided useful guidance for understanding the nature and nosology of tremulous movements in different isolated dystonia syndromes (focal, segmental, multifocal and generalized) [24]. The study found an overall tremor prevalence of 53.3%, and factors predicting dystonic tremor varied according to the criteria (Fahn’s vs. MDS 1998/2018) used to define them [1, 3, 20]. The study identified several important factors that significantly influenced the prevalence of tremor in dystonia. They included affected body regions, severity of dystonia, and differences in opinion among investigators. We set out to conceptualize a similar study with a comparable sample size, just focusing on CD. We studied the prevalence of neck tremor and manifestations of different types of tremor (irregular/jerky vs. regular/sinusoidal) in CD. The large number of cases and multi-center study design facilitated the identification of factors that influence the prevalence of neck tremor and importantly the ones that determine jerky versus regular tremor in CD. The results provide useful guidance for understanding the nature and nosology of tremulous neck movements in patients with CD.

## Methods

### Participants

Participants were recruited from 37 sites of the Dystonia Coalition, a part of the NIH Rare Diseases Clinical Research Network.^1^ Most sites are in North America (United States and Canada), four in Europe (France, Germany, Italy, United Kingdom) and one in Australia.

We received institutional approval from an ethical standards committee on human experimentation for any protocol using human patients. All participants in the study provided written informed consent. This study is not a clinical trial, hence public trials registry or clinical trial identifiers are not applicable.

Inclusion criteria stated that participants had to have a minimum of 18 years of age and a diagnosis of CD [25]. Any region of the body could be affected, alone or in various combinations (focal, segmental, multifocal, and generalized). Most cases were idiopathic, but a small fraction had associated known genetic etiologies [26]. The study excluded dystonia syndromes combined with other neurologic features (previously known as dystonia-plus syndromes or heredodegenerative dystonias), acquired dystonias (such as tardive syndromes or encephalitis), and functional (psychogenic) dystonia. Participants treated with botulinum toxin were not excluded, although all participants were enrolled when the movement disorder was apparent, which was typically at least 3 months following treatment, and never less than 2 months following treatment. Prior surgery for dystonia was not an exclusion criterion for the Dystonia Coalition cohort, but all such cases were excluded from this study to avoid inclusion of cases where surgery might result in atypical residual manifestations.

### Clinical assessment of dystonia and tremor

Clinical assessment of dystonia and tremor has been explained in detail in our previous report [24]. In summary, a standardized form was used to collect data [27]. Experts of movement disorders evaluated the cases, following a standardized and structured neurologic examination [27]. The Global Dystonia Rating Scale (GDRS) [28] was used to assess severity and body distribution of dystonia. The Essential Tremor Rating Assessment Scale (TETRAS) [29] was employed for the assessment of tremors. Tremor was classified as irregular or regular based on Fahn’s definition [30].

### Characteristics of participants with dystonia

A total of 3,117 patients with non-zero GDRS neck scores were included in this report. The average age at evaluation was 60.1 ± 12.3 years (median 61, range 18–92). The average age at dystonia onset was 46.3 ± 14.7 years (median 48, range 0–82), with an average illness duration of 13.8 ± 12.47 years (median 10, range 0–81). Women (*n* = 2,257) outnumbered men (*n* = 860) by a ratio of 2.6 to 1. Most were white (*n* = 2,892) while others were black (*n* = 119), Asian (*n* = 27), American Indian or Alaska Native (*n* = 17) or of other or unknown/unreported race (*n* = 62). In our cohort 2,696 patients were right-handed, 289 patients were left-handed, 95 patients were ambidextrous while handedness of 37 patients was unknown.

**FIGURE 2:**
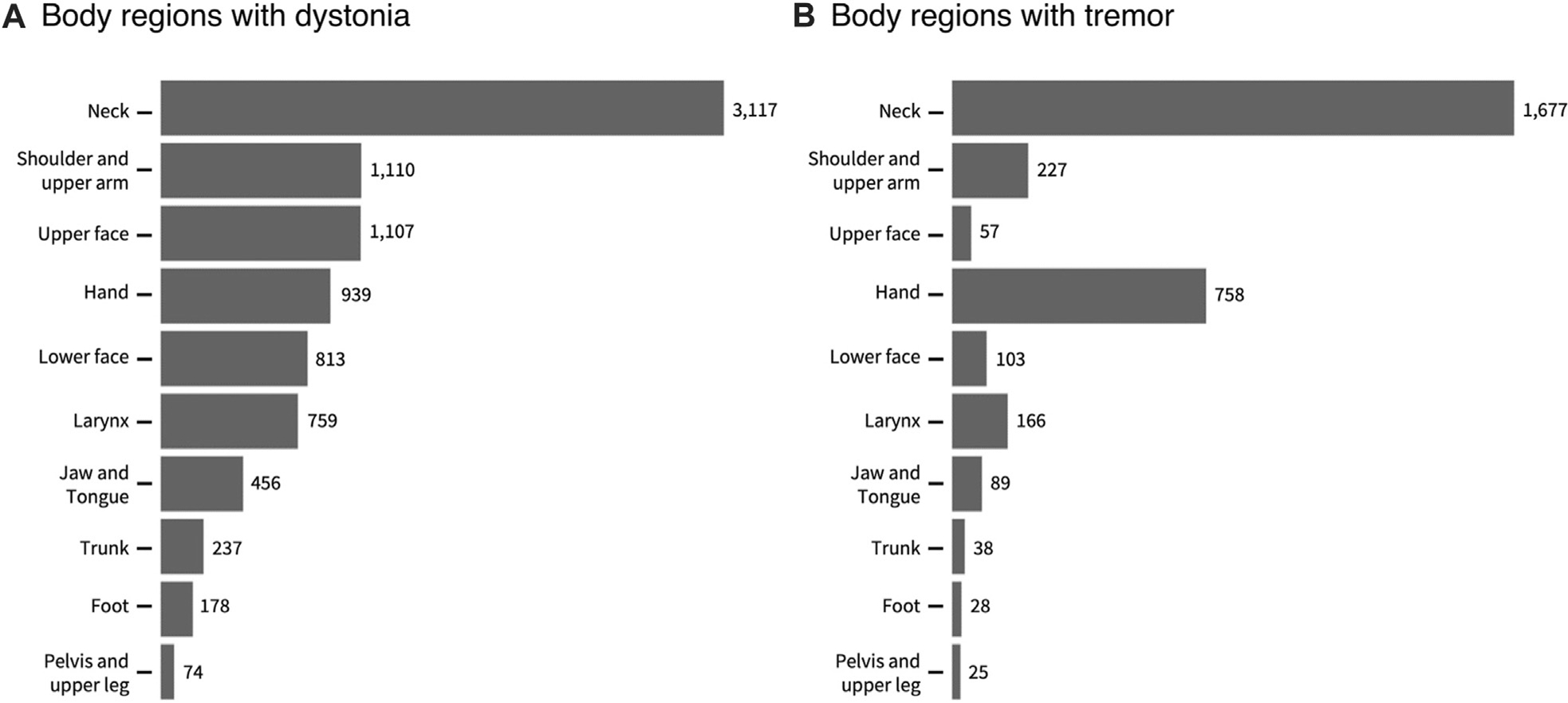
A The totals for individual body regions with dystonia **(A)** and tremor **(B)** in 3117 participants with cervical dystonia. The totals may sum up to more than the total number of participants because many participants had more than one body region affected. The numbers in the figures show the actual numbers of participants with each region affected.

Among 3,117 patients with CD, the neck dystonia was isolated in 1,791 but some had segmental dystonia (*n* = 681), multifocal dystonia (*n* = 160), generalized dystonia (*n* = 96), or hemidystonia (*n* = 10). Average dystonia severity as assessed with GDRS total score was 9.18 ± 7.88 (median 7, range 1–113) with a mean GDRS neck score 4.55 ± 2.16 (median 4, range 1–10). The distribution of body regions with dystonia and tremor can be seen in Figures 2A, B.

In this cohort of 3117 individuals with CD, the overall prevalence of any type of tremor (regular or irregular or both) in any body region was 60%. At total of 37.8% of the cohort had focal CD. Based on the highest non-neck GDRS score, 31.4% of the cohort had additional limb dystonia (upper and lower extremities combined, including shoulder). 20.14% also had cranial dystonia affecting upper and lower face, tongue, or jaw. 8.24% had laryngeal dystonia, and 2.41% had pelvis/trunk dystonia.

### Data analysis

Binomial logistic regression models with a logit link function were used to evaluate the clinical characteristics predictive of neck tremor and to determine the important features distinguishing neck tremor from no tremor. This analysis was also performed for female and male populations, separately, to test whether there are differences between men and women in the features related to neck tremor. Feature importance analyses were done using the Wald test (aka the Wald Chi-Squared Test) which was applied to each parameter of the model to test whether it has a significant contribution to the model. Clustering analyses were performed to identify cohort subgroups with common clinical features found significantly important in predicting the occurrence of neck tremor.

Binomial logistic regression models were also deployed to identify the important clinical characteristics associated with regular neck tremor in CD compared to the irregular type, as well as the ones related to regular neck tremor relative to no tremor. For a tremor case to be classified as “regular,” the patient had to have either no other body part affected with tremor, or if they had other body parts affected with tremor, they had to be of regular type. Similarly, for a tremor case to be classified as “irregular,” the patient had either no other body part with tremor, or other body parts affected with tremor also had irregular tremor type. The patients with mixed regular and irregular tremor were excluded from this analysis.

## Results

### Overall prevalence of neck tremor

To identify the important clinical characteristics associated with neck tremor in CD, we considered the patients with neck GDRS scores larger than zero. We aggregated the cases where dystonia was focal, multi-focal, segmental, or generalized (N = 3117). 18 records with incomplete information (one age, two dystonia duration, and 15 GDRS scores) were discarded from the analysis. The remaining complete records (N = 2,999) were included into an GLM analysis to examine the relationship between neck tremor (two levels for presence and absence of neck tremor) and patient attributes including age, duration of dystonia, total neck GDRS score, total non-neck GDRS score, race (three levels for white, black, and other), sex (two levels for male and female), recruitment site (30 sites, each having minimum 20 patients), handedness (four levels for ambidextrous, left, right, and unknown) and dystonia location (two levels, one level for focal CD with zero GDRS score in body parts other than the neck, and another level for non-focal CD). Continuous attributes (age, duration, neck, and non-neck GDRS scores) were standardized by subtracting the mean and dividing by the standard deviation. The GLM was a binomial logistic regression with a logit link function:

Necktremor(Y/N)~Age+Dystoniaduration+NeckGDRS+Non-neckGDRS+Dystoniatype+Race+Sex+Site+Handedness.

**FIGURE 3:**
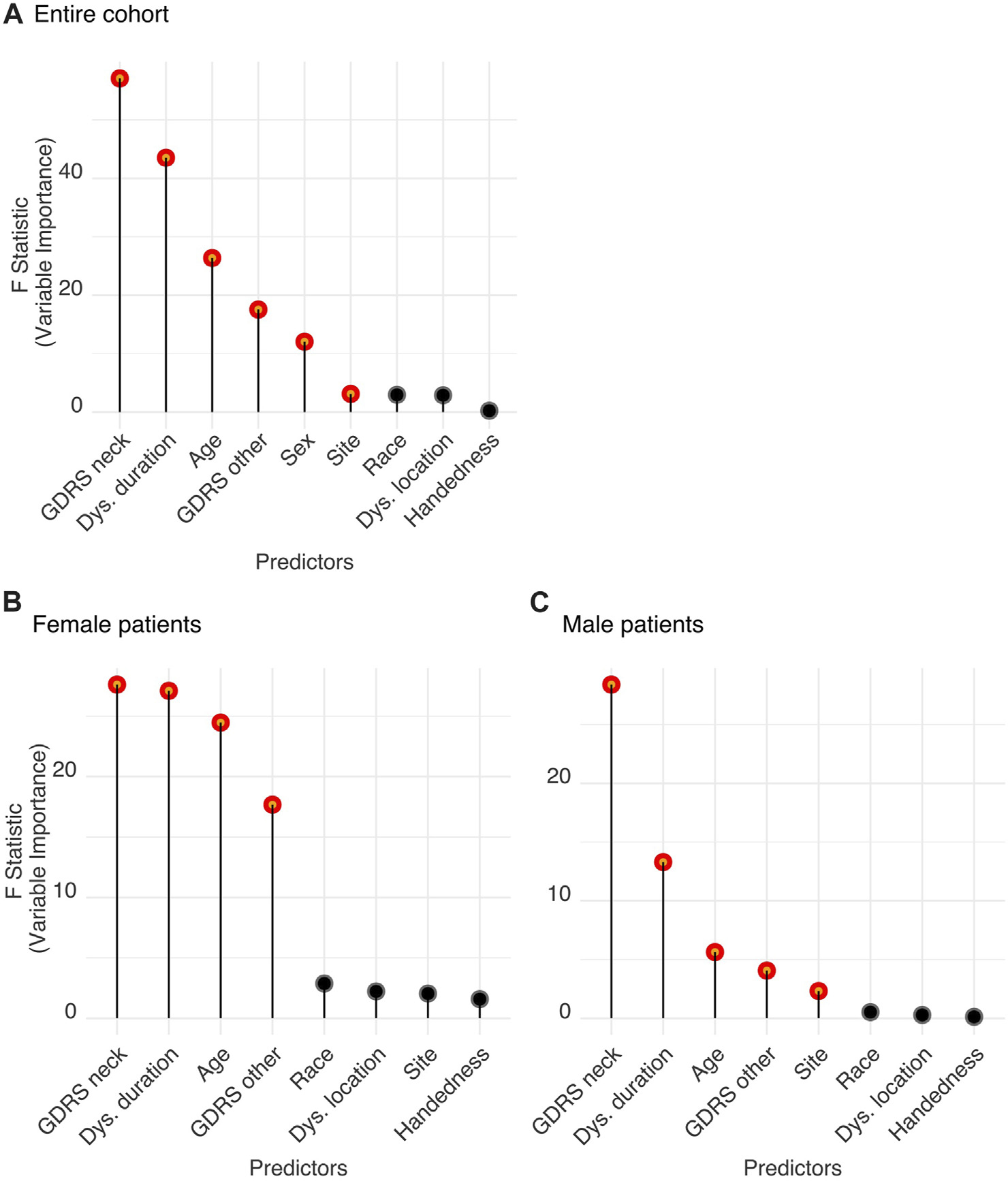
**(A)** Features relevant for neck tremor in the CD population. **(B,C)** Features relevant for neck tremor in **(B)** female and **(C)** male patients. Significant features predicting tremor were determined by Wald tests. Significant parameters (shown in red) are significantly different from zero and produce a statistically significant decline in the logistic regression model once removed. The impact of each parameter is estimated by the length of the line. Non-significant features are shown in black.

The logistic regression model with the listed predictors fitted significantly better than the null model (likelihood ratio test, Chi-squared = 350.32, *p* < 0.001). Analysis of variance for the model’s individual terms is summarized in Table 1 (standardized regression coefficients are reported for numerical predictors and odds ratios for categorical variables). Figure 3A depicts the significantly important features that distinguish between neck tremor and no neck tremor conditions considering the entire cohort with CD. We found that severity of neck dystonia as assessed with neck GDRS score was the most important patient characteristic predicting neck tremor (standardized coefficient = 0.318 (0.236–0.401), *p* < 0.001). High CD severity was related to increased likelihood of neck tremor. The next most important predictors of neck tremor were dystonia duration and age, which were also associated with increased neck tremor prevalence (duration: 0.285 (0.201–0.371), age: 0.218 (0.135–0.302), *p* < 0.001). The negative coefficient of non-neck GDRS (total score minus neck score) indicated that severity of dystonia in other parts of the body was associated with decreased likelihood of neck tremor. Sex was also significant in predicting neck tremor. Compared to females, males were 0.736 times less likely to have neck tremor, suggesting a heightened risk of neck tremor for female patients. Site, i.e., the investigator bias, was a significant, but the least important predictor of neck tremor prevalence. The comparison was made with the reference site, revealing the significant differences between six sites and the reference site with 52.06% neck tremor prevalence rate (see the odds ratios in Table 1). Race, dystonia location and handedness were not significant in predicting neck tremor (*p* > 0.05).

Additionally, we performed another logistic regression analysis to further examine the role of the second body part affected by dystonia in predicting the prevalence of neck tremor. We contrasted the following dystonia combinations to the isolated CD:

1. neck + cranial region (including face, tongue, and jaw),
2. neck + larynx,
3. neck + limbs (including upper and lower extremities),
4. neck + pelvis/trunk.

We found that CD with additional cranial symptoms significantly decreased the likelihood of neck tremor [OR (95% CI) = 0.632 (0.491–0.81), *p* < 0.001] while dystonia affecting the larynx in addition to the neck increased the likelihood of neck tremor [OR (95% CI) = 1.47 (1.056–2.058), *p* = 0.024]. Additional limb or pelvis/trunk dystonia did not have a significant influence on neck tremor (*p* > 0.05).

### Clustering of the cohort based on the features predicting neck tremor

A K-means clustering analysis was applied using the statistically significant features of the logistic regression analysis reported in the previous section. Although recruitment site was found significant, it was excluded from the clustering analysis with which we aimed to consider only phenotypically relevant patient characteristics associated with dystonia. The clustering algorithms (K-means as well as other Gower-distance based methods) were found to be sensitive to the only categorical variable in the feature set: sex. All algorithms first grouped the cohort into two groups based on sex. Hence, we performed two independent clustering analyses: one for female and another for male sub-cohort, to be able to more accurately distinguish the subgroups based on the other important neck tremor-predicting features. The four significant predictors of neck tremor: CD severity (measured by GDRS of neck), dystonia duration, age, and non-CD severity (measured by GDRS non-neck) were included in the clustering analysis (as shown in Figure 3B for female and Figure 3C male patient data and detailed in Table 1).

**FIGURE 4:**
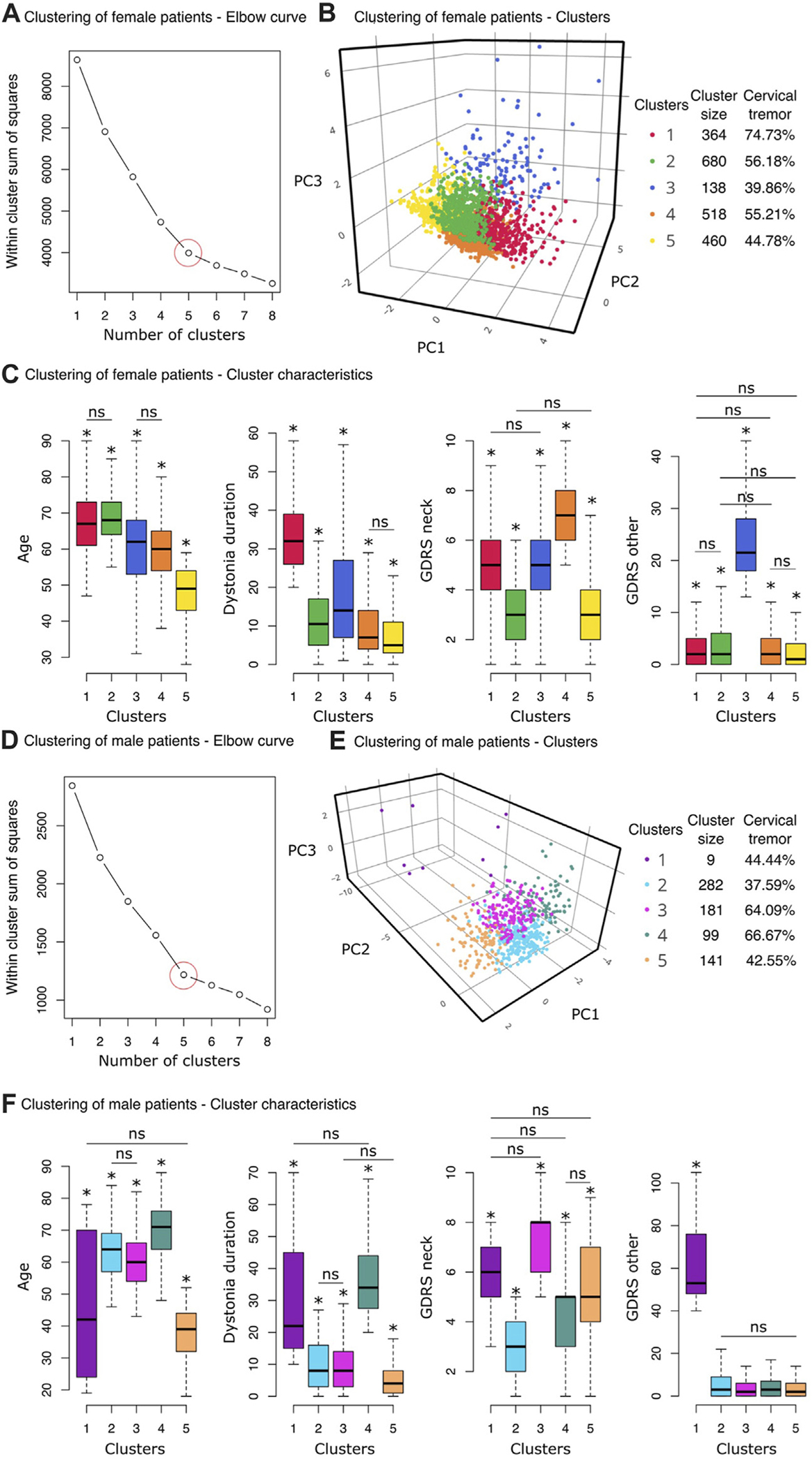
Clustering analyses results. **(A–C)** Clustering of female patients. **(A)** The Elbow method was used to find the optimum number of clusters, k. Within-cluster sum of squares (the sum of squared distance between each point and the centroid in a cluster) are plotted for a range of number of clusters (k = [1,8]). At k = 5, the slope of the graph changes, creating an elbow shape. This point was considered to be the optimal number of clusters for the female group. **(B)** The three-dimensional scatter plot displays the first three principal components of the five clusters detected by the k-means clustering algorithm (for interactive plot: https://chart-studio.plotly.com/~sinoscope/125). Cluster sizes and neck tremor prevalence rates of the clusters are shown in the legend. **(C)** Boxplots from left to right show age, dystonia duration, CD severity (GDRS neck) and non-CD severity (GDRS other) distributions of the 5 clusters. An asterisk above a box indicates a statistically significant pair-wise difference between that box and all the others except for the pairs marked with “ns.” **(D–F)** Clustering of male patients. **(D)** k = 5, where the graph makes an elbow shape, was considered to be the optimal number of clusters for the male cohort. **(E)** The three-dimensional scatter plot displays the first three principal components of the four clusters detected by the k-means clustering algorithm (for interactive plot: https://chart-studio.plotly.com/~sinoscope/131). Neck tremor prevalence rates of the clusters are shown in the legend. **(F)** Boxplots (from left to right) demonstrate age, dystonia duration, CD severity (GDRS neck) and non-CD severity (GDRS other) distributions of the 5 clusters. An asterisk above a box indicates a statistically significant pair-wise difference between that box and all the others except for the pairs marked with “ns.”

The elbow method, which is an optimization method that finds the smallest number of clusters (k) accounting for the largest amount of variation in the data, was applied to the female subcohort. The optimum number of distinct groups appeared to be five (Figure 4A, circled in red). A three-dimensional scatterplot of the first three principal components (Figure 4B) illustrates the five clusters with prevalence of neck tremor varying from 39.86% (Cluster 3) to 74.73% (Cluster 1). The characteristics of the clusters are summarized in Table 2.

We carried out pairwise comparisons between the clusters for the four features used in the clustering of female patients (mean and standard deviations are in Table 2, pairwise comparison statistics in Supplemental Material). The difference was considered significant at *p* < 0.001 after correcting for multiple comparisons following Tukey’s method. Asterisks in Figure 4C demonstrate the significant difference of the designated cluster from the other clusters except the pairs marked with “ns” for statistically non-significant difference. Cluster 1, which has the highest neck tremor prevalence (74.73%) among female patients, was distinguished with the longest dystonia duration (Mean ± SD: 34.16 ± 10.01 years) (Figure 4C). However, this cluster, which contained the oldest female patients along with Cluster 2, had only the second highest average CD severity (GDRS neck score: 5.06 ± 3.67). On the other hand, Cluster 4 with 55.21% neck tremor prevalence had significantly the highest CD severity among female patients (GDRS neck score: 6.79 ± 3.73). Cluster 5 was distinct from the other clusters by its highest non-CD severity (GDRS other: 24.79 ± 2.12). All other pairs were statistically similar in this feature. The youngest female patients (47.49 ± 8.64 years) were clustered into Cluster 5 which had 44.78% neck tremor prevalence (Figure 4C). The other characteristics of this cluster also took the lowest values among the other clusters. Cluster 2, with a slightly higher neck tremor rate of 56.18%, shared the lowest rank in CD severity with Cluster 5 (3.01 ± 3.83 and 3.23 ± 3.93) while having significantly higher average age than Cluster 5 (68.45 ± 6.39 vs. 47.49 ± 8.64).

For male patients, the elbow method also revealed five clusters as the optimum number of distinct subgroups (Figure 4D, circled in red). A three-dimensional scatterplot of the first three principal components (Figure 4E) illustrates the five clusters with prevalence of neck tremor varying from 37.59% (Cluster 2) to 66.67% (Cluster 4). The characteristics of the clusters are summarized in Table 2.

Pairwise comparisons were carried out between the clusters for the four features used in the clustering of male patients (mean and standard deviations are in Table 2, pairwise comparison statistics in Supplemental Material). The cluster with the minimum neck tremor prevalence rate of 37.59%, Cluster 2, contained the male patients with the minimum CD severity (3.04 ± 1.31) as well as the lowest dystonia duration (9.75 ± 7.18, together with Cluster 3) and lowest non-CD severity (5.60 ± 6.40, together with Clusters 3, 4, and 5) (Figure 4F). On the other hand, Cluster 4 had the highest neck tremor prevalence rate of 66.67% with the highest age (69.31 ± 9.90) and dystonia duration (36.58 ± 11.14 years) (together with Cluster 1) but not the highest cervical or non-CD severity (Figure 4F). Cluster 1, which has a neck tremor prevalence rate of 44.44% among male patients, was distinguished with the highest non-CD severity (62.89 ± 23.50) (Figure 4F). Cluster 3 with a neck tremor rate of 64.09% contained the male patients with the highest CD severity scores (7.48 ± 1.25, together with Cluster 1). Youngest patients (37.72 ± 7.81, together with Cluster 1) with lowest dystonia durations (5.99 ± 6.25, together with Cluster 3) were grouped into Cluster 5, which had a neck tremor rate 42.55% (Figure 4F).

### Neck tremor regularity

#### Regular vs. irregular neck tremor

To identify the important clinical characteristics associated with regular neck tremor in CD compared to the irregular type, we included 1,367 patients from the cohort who have CD as well as neck tremor. These patients had a complete set of clinical features available and were recruited in sites with more than 20 patients. The percentages of patients with regular and irregular neck tremor were 25.24% and 74.76%, respectively.

The imbalance between the number of samples with regular and irregular neck tremor cases (1,022 irregular vs. 345 regular cases) may bias the logistic regression model towards the majority group. To overcome this imbalance, we drew a sample data set from the irregular neck tremor group with the size comparable to the size of the regular neck tremor group. This under-sampling process was carried out with stratification on the entire set of variables to make sure feature distributions were preserved (confirmed visually as well as by two-sample t-tests with *p* > 0.05). The resulting data set had a size of 539 patients (257 irregular vs. 282 regular cases). A GLM analysis was used to predict the relationship between regularity of neck tremor (compared to irregularity) and patient attributes including age, duration of dystonia, CD severity (GDRS neck), non-CD severity (GDRS other), race (three levels for white, black, and other), sex (two levels for male and female), recruitment site (11 sites), and dystonia location (two levels, one level for isolated CD and another level for non-isolated CD). Continuous attributes were standardized. The GLM was a binomial logistic regression with a logit link function:

Necktremortype(Regular/Irregular)~Age+Dystoniaduration+GDRSneck+GDRSother+Dystonialocation+Race+Sex+Site

**FIGURE 5:**
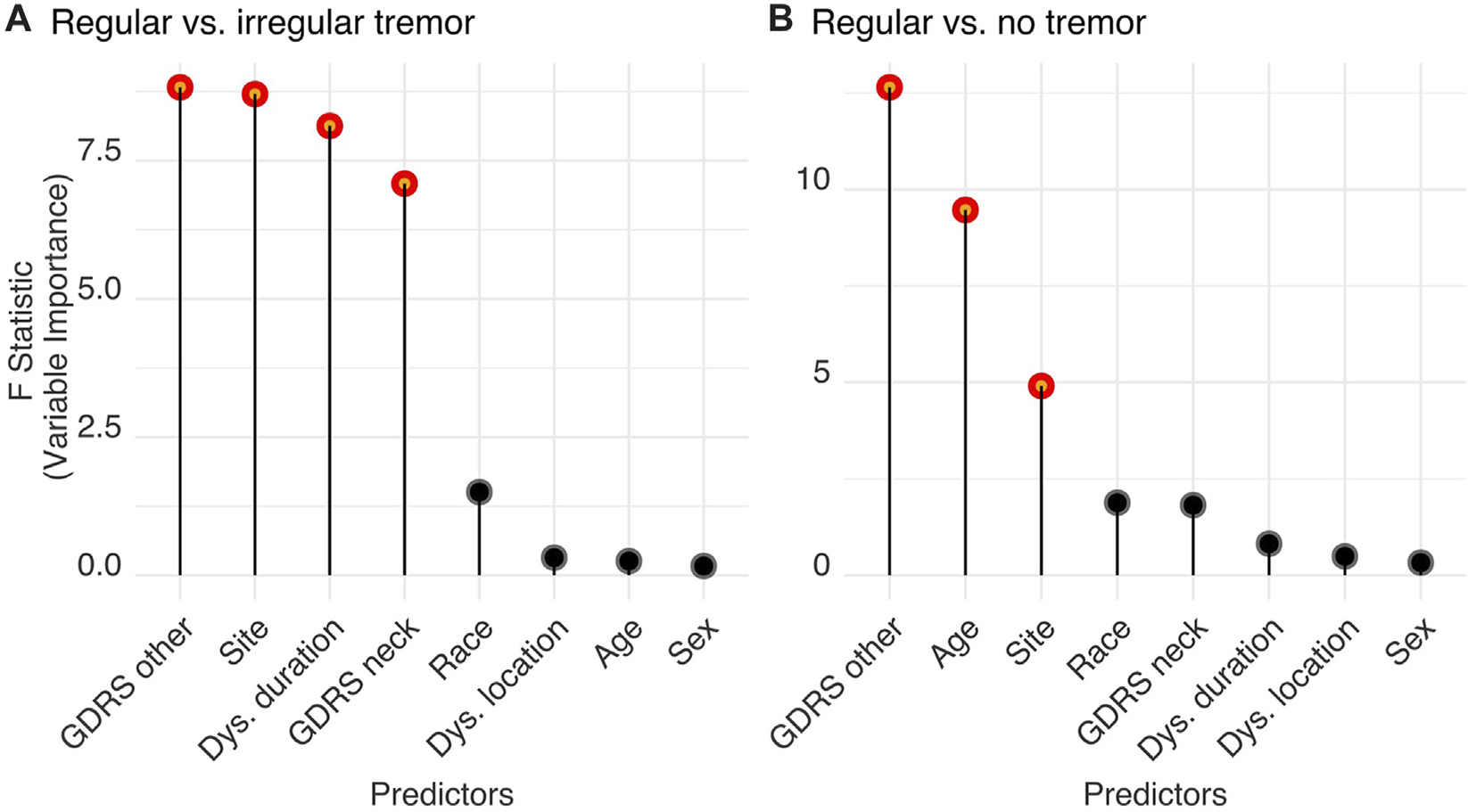
Features significantly distinguish **(A)** regular from irregular neck tremor, and **(B)** regular neck tremor from no tremor. Significant features (shown in red) are significantly different from zero as tested using Wald tests and produce a statistically significant decline in the logistic regression model once removed. The impact of each parameter is estimated by the length of the line. Non-significant features are shown in black.

The logistic regression model with the listed predictors fitted data significantly better than the null model (likelihood ratio test, Chi^2^ = 180.71, *p* < 0.001). Analysis of variance for the model’s individual terms is summarized in Table 3 (standardized regression coefficients are reported for numerical predictors and odds ratios for categorical variables). Important features are also displayed in Figure 5A with red. We found that non-CD severity was the most important patient characteristic predicting tremor regularity (standardized coefficient: 0.498 (−0.853 to −0.193), *p* = 0.003). High non-CD severity was related to decreased likelihood of regular neck tremor (i.e., increased likelihood of irregularity). The next most important predictor of neck tremor regularity was site. Out of 10 sites, two sites were associated with increased neck tremor regularity (one 2.519 (1.065–6.078) times and the other 15.782 (7.139–36.468) times) and one site with decreased neck tremor regularity (0.078 (0.012–0.293) times) compared the reference site with 52.06% tremor prevalence rate (the reference site used in the tremor vs. no tremor analysis) (Table 3). Dystonia duration [−0.335 (−0.570 to −0.108), *p* = 0.004] and CD severity [−0.324 (−0.567 to −0.088), *p* = 0.008] were the other features significantly distinguishing regular from irregular neck tremor. High values were associated with increased irregularity in neck tremor. Race, dystonia location, age, and sex were not found significant in differentiating regular from irregular neck tremor (*p* > 0.05).

#### Regular versus no neck tremor

We also investigated the important clinical characteristics associated with regular neck tremor in CD compared to the condition where no body part is affected with tremor. We included 1,531 patients from the cohort with either regular neck tremor (*n* = 1,186) or without *any* tremor (*n* = 345). These patients had a complete set of clinical features available and were recruited in sites with at least 20 patients. The percentage of patients with regular neck tremor and without tremor were 22.53% and 77.47% respectively.

Analogous to the previous analysis, we attempted to remove a potential bias that may emerge from the imbalance in the data by drawing a sample set from the no tremor group with the size comparable to the size of the regular neck tremor group. Sampling was done with stratification on the entire set of included variables to make sure feature distributions were protected (confirmed visually and by two-sample t-tests with *p* > 0.05). The resulting data set had 576 patients (284 no tremor vs. 292 regular cases). Age, duration of dystonia, CD severity (GDRS neck), non-CD severity (GDRS other), race (three levels for white, black, and other), sex (two levels for male and female), recruitment site (12 sites), and dystonia location (two levels, one level for isolated and another level for non-isolated CD) were included in a GLM model after the standardization of continuous attributes. The GLM was a binomial logistic regression with a logit link function:

Necktremortype(Regular/NoTremor)~Age+Dystoniaduration+GDRSneck+GDRSother+Dystonialocation+Race+Sex+Site

The logistic regression model with the listed predictors fitted significantly better than a null model (likelihood ratio test, Chisq = 148.67, *p* < 0.001). Results are summarized in Table 3; Figure 5B. Similar to the previous analysis, non-CD severity was again the most important patient characteristic distinguishing regular neck tremor from no tremor [−0.523 (−0.827 to −0.248), *p* < 0.001]. High non-CD severity was related to decreased likelihood of regular neck tremor (or increased likelihood of no neck tremor). The second most important feature predicting regular neck tremor with respect to no tremor condition was age—higher age predicted increased likelihood of regular neck tremor [0.332 (0.123–0.547), *p* = 0.002]. Site was also found significantly important for regular neck tremor. Out of 11 sites, two sites had significantly more patients with regular neck tremor than patients with no tremor compared to the reference site with 52.06% tremor prevalence rate [one site 2.402 (1.192–4.920) times and the other site 4.644 (2.412–9.108) times]. One site had significantly less regular neck tremor cases than no tremor in contrast to the reference site [0.189 (0.028–0.730) times]. Race, CD severity, dystonia duration, dystonia location, and sex were not found significant in differentiating regular irregular neck tremor from no tremor (*p* > 0.05).

## Discussion

This is a prospective, multi-center investigation involving sites from North America, Europe, and Asia, examining the prevalence and semiology of clinically apparent neck tremors in patients with CD. Tremor is common in dystonia, and it is highly prevalent in focal forms such as CD [24]. There is a varying co-prevalence rate of tremor and dystonia and the rate depends on factors such as the body regions affected with dystonia, age and duration of dystonia, severity of dystonia, and importantly how the tremor is defined and the investigators’ threshold on labeling the given movement as “tremor” [24]. Our study found that severity of CD, increased dystonia duration and age, as well as female sex positively correlate with presence of neck tremor. Indeed neck tremor at disease onset represents a clinically distinguishable subtype of CD affecting predominantly older women, with worse ataxia and milder dystonia than the non-tremulous dystonic phenotype [31–33]. We also found that neck tremor is less likely to be present in CD if dystonia also exists elsewhere other than the neck. Increased severity of neck dystonia is not only associated with presence of neck tremor, but also with irregular tremor type. Here we address these findings and explain how they may facilitate the understanding of the dystonia-tremor relationship.

### Clinical factors relevant to the prevalence of neck tremor in CD

The co-prevalence of tremor and dystonia have specific patterns and are influenced by several factors. For example, limb essential tremor is commonly associated with dystonia of head/neck and voice [34–37]. In line with prior studies [38, 39], we found that the prevalence of neck tremor depended on other factors such as age, as well as the duration and the severity of CD. We found robust variability in prevalence of tremor depending on how it was diagnosed. Such variability was also present in the very common CD [24]. It is also likely that threshold for diagnosing tremor varies across different investigators. The variation is even more robustly present for dystonic tremor, independent of the definition followed for diagnosis [40]. These between-investigator differences may explain the discrepancies among recent studies that included very similar cohorts of dystonia patients, using the same definitions for tremor [38, 39].

This study presents analytical results from the largest cohort of systematically evaluated CD patients available to date. The cohort is multi-center, involving multiple races, and ethnicities. The design of this study suggests that the conclusions are not influenced by issues related to small cohort size, non-representative subtypes of dystonia or tremor, or investigator bias for diagnosis and evaluations. Nevertheless, we also acknowledge some limitations of this study. The major limitation is the dependency of neck tremor detection threshold on clinical evaluation. There are more sensitive methods for detecting tremor including objective techniques such as kinematic tools [41–43] or electromyography [44–46]. These methods are much more sensitive than clinical examination alone [47]. Therefore, it is possible that the actual neck tremor prevalence is much higher than clinically estimated in this study.

Another limitation is related to the ongoing controversy over the definition of “dystonic tremor” and the lack of systematic and consistent evaluation for a “null point,” which is characteristic feature of dystonic tremor [2]. Our design considered both commonly used definitions independently. It focused on the key differences in the diagnostic criteria such as regularity, jerkiness, and concurrence with dystonia. We found that despite the evidence-based approach, varying opinions among investigators influenced the impressions for labeling a tremor as “irregular” or “jerky.” Varying opinions also influenced the diagnostic threshold for diagnosing a movement as “tremor” or labeling tremulous movements in body regions concordant with dystonia. In these situations, instrumented measures may better discriminate the characteristics of tremor [42, 43] and could be useful to determine the true prevalence of each type.

The third limitation was that the study relied on data recorded by the investigators at a recruitment site without the verification of an independent second evaluation. Although all investigators used the same protocol for evaluation, thresholds for diagnosing tremor clearly vary among investigators. Future studies may benefit from more objective and independent methods. Despite these weaknesses, the results provide the most comprehensive picture of tremor in subjects with CD currently available.

### Biological factors relevant to neck tremor in CD

There is a high prevalence of tremor in CD, and increasing evidence suggests overlapping biological mechanisms. For instance, it is a common observation that an individual who has isolated tremor for many years can present with dystonia movements in same or another body part [48–50]. It is also possible that patients who have “pure” dystonia for a long time can present with emergence of tremor [49, 50]. There are studies showing common anatomical substrates for tremor and dystonia. Common tremor syndromes result from abnormal functioning of cerebellar circuits [51, 52]. The cerebellum has an important role in motor network causing dystonia [53–56], and particularly CD. A PET study using fluoro-deoxyglucose revealed multiple significant abnormalities when comparing patients with dystonia or essential tremor with normal controls [57]. These abnormalities overlap considerably among the dystonia and tremor groups. Patients with tremor [58] or dystonia [59, 60] have similar histopathological abnormalities affecting the cerebellum, such as loss of Purkinje neurons and torpedo inclusion bodies. A recent study identified physiological similarities in pallidal single unit responses in patients who have jerky, “dystonic tremor” and torsion neck dystonia [22].

Our study also found differences in predictors of tremor and dystonic tremor in male versus female patients. Such differences depict sex-specific distinctions in the pathophysiology of tremor and dystonia. Genetic and family studies provide further insights into shared biological mechanisms of dystonia and tremor [35, 61–64]. Although some such cases may represent misdiagnoses, it seems more likely that the occurrence of tremor syndromes with “dystonia” genes represents the sometimes highly varied pleiomorphic clinical phenotypes associated with monogenic and oligogenic variants. Our study found differences in the predictors of tremor and dystonic tremor in male versus female patients. These findings point toward sex-specific differences in the pathophysiology of tremor and dystonia. In sum, we evaluated a large dystonia cohort and identified the most relevant clinical features that can predict concurrent tremor and its irregularity. Our results provide a more complete description of CD and may help improve care in CD and other forms of dystonia.

## Figures and Tables

**TABLE 1:** Summary of the results of the logistic regression models applied to the entire cohort (N = 2,999, top), to the female group (N = 2,115, middle) and to the male group (N = 712, bottom) formed by patients recruited by sites with equal or more than 20 patients. Significant factors are listed. Standardized coefficients are reported for continuous factors and odds ratios for categorical factors (with 95% confidence intervals).

Predictor	Std. Coefficient (95%)	Odds ratio (95% CI)	*p*-Value

***Binomial multiple logistic regression analysis of Neck Tremor vs. No Neck Tremor—Entire Cohort***			

GDRS neck	0.318 (0.236–0.401)		<0.001*

Dystonia duration	0.285 (0.201–0.371)		<0.001*

Age	0.218 (0.135–0.302)		<0.001*

GDRS other	−0.218 (−0.322–−0.118)		<0.001*

Dystonia location (Ref: non-focal CD)		1.183 (0.974–1.436)	0.090

**Race (Ref: White)**			

Black		0.661 (0.435–0.992)	0.048*
Other		0.705 (0.444–1.108)	0.133

Sex (Ref: Female)		0.736 (0.619–0.875)	<0.001*

**Handedness (Ref: Right)**			

Ambidextrous		0.922 (0.593–1.437)	0.718
Left		0.906 (0.695–1.182)	0.4665
Unknown		0.904 (0.441–1.862)	0.7827

**Site (Ref: Median site with 52.06% prevalence rate)**			

Site 18		2.601 (1.320–5.464)	0.008*
Site 30		0.032 (0.002–0.158)	<0.001*
Site 27		0.254 (0.083–0.645)	0.008*
Site 29		0.228 (0.083–0.567)	0.002*
Site 19		0.493 (0.243–0.971)	0.044*
Site 20		0.201 (0.076–0.469)	<0.001*

***Binomial multiple logistic regression analysis of Neck Tremor vs. No Tremor—Female Patients***			

GDRS neck score	0.274 (0.178–0.372)		<0.001*

Dystonia duration	0.267 (.169–0.367)		<0.001*

Age	0.244 (.148–0.341)		<0.001*

GDRS other	−0.260 (−.379–−0.146)		<0.001*

Dystonia location (Ref: non-focal CD)		1.227 (0.976–1.542)	0.080

**Race (Ref: White)**			

Black		0.593 (0.364–0.951)	0.032*
Other		0.672 (0.379–1.174)	0.1663

**Handedness (Ref: Right)**			

Ambidextrous		0.954 (0.553–1.657)	0.867
Left		0.822 (0.600–1.128)	0.2244
Unknown		0.447 (0.179–1.066)	0.0736

**Site (Ref: Median site with 52.06% prevalence rate)**			

Site 18		3.016 (1.439–6.842)	0.005*
Site 26		5.743 (1.590–36.852)	0.022*
Site 10		1.805 (1.038–3.203)	0.039*
Site 30		0.060 (0.003–0.329)	0.008*
Site 27		0.350 (0.112–0.919)	0.046*
Site 4		1.683 (1.115–2.552)	0.014*
Site 3		1.482 (1.018–2.166)	0.041*

***Binomial multiple logistic regression analysis of Neck tremor vs. No Neck Tremor—Male Patients***			

GDRS neck score	.480 (0.306–0.660)		<0.001*

Dystonia duration	.339 (0.159–0.525)		<0.001*

Age	.216 (0.039–0.396)		0.018*

GDRS other	−.229 (−0.468–−0.019)		0.044*

Dystonia location (Ref: non-focal CD)		1.115 (0.742–1.672)	0.598

**Race (Ref: White)**			

Black		1.135 (0.469–2.684)	0.775
Other		0.664 (0.279–1.490)	0.3338

**Handedness (Ref: Right)**			

Ambidextrous		1.032 (0.460–2.314)	0.9392
Left		1.139 (0.676–1.915)	0.6241

**Site (Ref: Median site with 52.06% prevalence rate)**			

Site 8		0.210 (0.089–0.469)	<0.001*
Site 7		0.418 (0.185–0.928)	0.033*
Site 9		0.281 (0.113–0.658)	0.004*
Site 6		0.305 (0.138–0.656)	0.003*
Site 17		0.332 (0.116–0.888)	0.032*
Site 5		0.390 (0.172–0.868)	0.022*
Site 19		0.179 (0.052–0.533)	0.003*

**TABLE 2:** Summary of the characteristics of the clusters formed by a K-means clustering algorithm applied to female and male patients. There were five optimum clusters for each of these populations with meaningfully distinctive clinical features. Values represent the mean ± standard deviation. Sample sizes and neck tremor rates (within cluster, in percentages) are also noted for each cluster.

Cluster characteristics - female patients						
Clusters	Size	Neck tremor prevalence (%)	Age	Dystonia duration	CD severity (GDRS neck)	Other dystonia severity (GDRS other)
1	364	74.73	67.20 ± 9.10	34.16 ± 10.01	5.06 ± 3.67	2.97 ± 1.67
2	680	56.18	68.45 ± 6.39	11.40 ± 7.57	3.01 ± 3.83	3.41 ± 1.19
3	138	39.86	59.77 ± 13.10	17.92 ± 14.53	5.09 ± 10.86	24.79 ± 2.12
4	518	55.21	59.43 ± 8.74	9.16 ± 6.74	6.79 ± 3.73	2.95 ± 1.19
5	460	44.78	47.49 ± 8.64	7.64 ± 6.57	3.23 ± 3.93	2.72 ± 1.34
Cluster characteristics—Male Patients						
Clusters	Size	Neck tremor prevalence (%)	Age	Dystonia duration	CD severity (GDRS neck)	Other dystonia severity (GDRS other)
1	9	44.44	45.22 ± 23.93	32.22 ± 22.09	5.89 ± 1.76	62.89 ± 23.50
2	282	37.59	63.55 ± 8.63	9.75 ± 7.18	3.04 ± 1.31	5.60 ± 6.40
3	181	64.09	60.52 ± 8.18	9.43 ± 7.34	7.48 ± 1.25	3.39 ± 4.07
4	99	66.67	69.31 ± 9.90	36.58 ± 11.14	4.48 ± 1.76	5.09 ± 6.01
5	141	42.55	37.72 ± 7.81	5.99 ± 6.25	4.99 ± 2.02	4.10 ± 6.26

**TABLE 3:** Summary of the results of the logistic regression models for factors differentiating regular from irregular tremor (top table) and regular from no tremor (bottom table). Significant factors are listed. Standardized coefficients are reported for continuous factors and odds ratios for categorical factors (with 95% confidence intervals).

Binomial multiple logistic regression analysis of regular vs. Irregular neck tremor			

Predictor	Std. Coefficient (95%)	Odds ratio (95% CI)	*p*-Value

GDRS neck	−0.324 (−0.567–−0.088)		0.008*

Dystonia duration	−0.335 (−0.570–−0.108)		0.004*

Age	0.057 (−0.163–0.279)		0.612

GDRS other	−0.498 (−0.853–−0.193)		0.003*

Dystonia location (Ref: Non-isolated)		0.856 (0.496–1.458)	0.572

**Race (Ref: White)**			

Black		0.116 (0.005–0.972)	0.088
Other		0.728 (0.125–4.049)	0.715

Sex (Ref: Female)		1.109 (0.678–1.816)	0.681

**Site (Ref: median site with 52.06% prevalence rate)**			

Site 4		0.078 (0.012–0.293)	0.001*
Site 6		2.519 (1.065–6.078)	0.037
Site 2		15.782 (7.139–36.468)	<0.001*

**Binomial multiple logistic regression analysis of Regular vs. No Neck tremor**			

Predictor	Std. Coefficient (95%)	Odds Ratio (95% CI)	*p*-value

GDRS neck	0.141 (−0.064–0.347)		0.177

Dystonia duration	0.095 (−0.110–0.302)		0.366

Age	0.332 (0.123–0.547)		0.002*

GDRS other	−0.523 (−0.827–−0.248)		<0.001*

Dystonia location (Ref: Non-isolated)		0.831 (0.492–1.389)	0.483

**Race (Ref: White)**			

Black		0.215 (0.0319–0.853)	0.054
Other		0.824 (0.189–3.214)	0.784

Sex (Ref: Female)		0.881 (0.571–1.360)	0.566

**Site (Ref: median site with 52.06% prevalence rate)**			

Site 4		0.189 (0.028–0.730)	0.034*
Site 3		2.402 (1.192–4.920)	0.015*
Site 2		4.644 (2.412–9.108)	<0.001*

## Data Availability

The original contributions presented in the study are included in the article/Supplementary material, further inquiries can be directed to the corresponding author.

## Footnotes

1
rarediseasesnetwork.org/cms/dystonia
